# Utility of Novel Cardiorenal Biomarkers in the Prediction and Early Detection of Congestive Kidney Injury Following Cardiac Surgery

**DOI:** 10.3390/jcm7120540

**Published:** 2018-12-12

**Authors:** Jason G. E. Zelt, Lisa M. Mielniczuk, Peter P. Liu, Jean-Yves Dupuis, Sharon Chih, Ayub Akbari, Louise Y. Sun

**Affiliations:** 1 Department of Cellular and Molecular Medicine, Faculty of Medicine, University of Ottawa, Ottawa, ON K1H8M5, Canada; jzelt@ottawaheart.ca (J.G.E.Z.); LMielniczuk@ottawaheart.ca (L.M.M.); 2 Division of Cardiology, Department of Medicine, University of Ottawa Heart Institute, 40 Ruskin Street Ottawa, ON K1Y 4W7, Canada; pliu@ottawaheart.ca (P.P.L.); schih@ottawaheart.ca (S.C.); 3 Division of Nephrology, Department of Medicine, The Ottawa Hospital, 1967 Riverside Dr., Ottawa, ON K1H 7W9, Canada; aakbari@toh.ca; 4 Division of Cardiac Anesthesiology, Department of Anesthesiology and Pain Medicine, University of Ottawa Heart Institute, 40 Ruskin Street, Ottawa, ON K1Y 4W7, Canada; jydupuis@ottawaheart.ca; 5 School of Epidemiology and Public Health, University of Ottawa, 600 Peter Morand Crescent, Ottawa, ON K1G 5Z3, Canada

**Keywords:** cardiac surgery, biomarkers, right heart failure, congestive acute kidney injury, venous congestion

## Abstract

Acute Kidney Injury (AKI) in the context of right ventricular failure (RVF) is thought to be largely congestive in nature. This study assessed the utility of biomarkers high sensitivity cardiac troponin T (hs-cTnT), N-Terminal Pro-B-Type Natriuretic Peptide (NT-proBNP), and neutrophil gelatinase-associated lipocalin (NGAL) for prediction and early detection of congestive AKI (c-AKI) following cardiac surgery. This prospective nested case-control study recruited 350 consecutive patients undergoing elective cardiac surgery requiring cardiopulmonary bypass. Cases were patients who developed (1) AKI (2) new or worsening RVF, or (3) c-AKI. Controls were patients free of these complications. Biomarker levels were measured at baseline after anesthesia induction and immediately postoperatively. Patients with c-AKI had increased mean duration of mechanical ventilation and length of stay in hospital and in the intensive care unit (*p* < 0.01). For prediction of c-AKI, baseline NT-proBNP yielded an area under the curve (AUC) of 0.74 (95% CI, 0.60–0.89). For early detection of c-AKI, postoperative NT-proBNP yielded an AUC of 0.78 (0.66–0.91), postoperative hs-cTnT yielded an AUC of 0.75 (0.58–0.92), and ∆hs-cTnT yielded an AUC of 0.80 (0.64–0.96). The addition of baseline creatinine to ∆hs-cTnT improved the AUC to 0.87 (0.76–0.99), and addition of diabetes improved the AUC to 0.93 (0.88–0.99). Δhs-cTnT alone, or in combination with baseline creatinine or diabetes, detects c-AKI with high accuracy following cardiac surgery.

## 1. Introduction

Right ventricular failure (RVF) is associated with significant morbidity and mortality following cardiac surgery [1,2]. Acute RVF is present in up to 50% of patients with postoperative hemodynamic instability [3] and is associated with difficult separation from cardiopulmonary bypass (CPB) [4], up to 75% increase in operative mortality as well as poor late survival [5,6]. Acute kidney injury (AKI) is a common sequela of RVF, which further complicates the management of RVF and portends a worsening prognosis. AKI occurs in up to 30% of cardiac surgical patients, of whom 1–2% require renal replacement therapy [7,8]. Management of perioperative AKI is primarily focused on maintenance of renal perfusion pressure and treatment of hypovolemia [9,10]. However, in the presence of RVF, AKI worsens with fluid administration and improves only with fluid removal [2]. This congestive form of AKI (c-AKI) is both difficult to diagnose and treat, especially as the cause of c-AKI is often not readily apparent and it is difficult to diagnose using current clinical criteria. Current diagnosis of AKI relies on measures of glomerular filtration such as serum creatinine, which is insensitive and lags behind actual renal injury; leading to delayed diagnosis and unfavourable outcomes [11,12]. Hemodilution, especially during the immediate postoperative period, may further confound creatinine-based diagnosis of AKI [13]. The paucity of early predictive biomarkers is one purported reason for the failure of recent prevention and treatment clinical trials for cardiac surgery induced AKI [14].

The pathogenesis of cardiac surgery induced AKI is multifactorial including many interrelated, and largely non-modifiable, injury pathways. However, venous congestion-induced AKI may potentially be reversed through diuretic and vasodilator based preventative and early treatment strategies. Contrary to popular belief [15], central venous pressure (CVP) is a poor indicator of circulating blood volume and fluid responsiveness [16,17]. Hence, there remains a need for better identification of patients who are most likely to benefit from early decongestive therapy.

Cardiac and renal biomarkers such as high sensitivity cardiac troponin T (hs-cTnT), N-Terminal Pro-B-Type Natriuretic Peptide (NT-proBNP), and neutrophil gelatinase-associated lipocalin (NGAL) are excellent prognosticators in patients with RVF and/or AKI [18,19,20,21,22,23,24,25]. These biomarkers provide direct cellular insight into cardiorenal physiology and may enable early identification of patients who are already in a congestive state prior to development of clinical consequences of venous congestion. However, the timing and pattern of release of these biomarkers are unknown in the setting of c-AKI with an acutely failing right ventricle (RV). An in-depth understanding of these biomarker patterns may enable identification of high-risk patients for intensive monitoring and early treatment. The objective of this study was to evaluate the utility of NT-proBNP, hs-cTnT, and NGAL for prediction and early detection of c-AKI following cardiac surgery. 

## 2. Methods

The research ethics board of the University of Ottawa Heart Institute (UOHI) approved this prospective nested case-control study (protocol #: 2015049401H). Written informed consent was obtained from all participants prior to enrolment.

### 2.1. Patients

We recruited 350 consecutive consenting patients aged 18 years or older, who underwent major elective cardiac surgery requiring CPB at UOHI between 29 September 2015 and 21 February 2017. Exclusion criteria included end stage renal disease (glomerular filtration rate [GFR] <15 mL/min or dialysis dependence), history of renal transplantation, solitary kidney, emergent operative status, off pump procedures, procedures involving circulatory arrest, heart transplantation, and left ventricular assist device implantation.

### 2.2. Outcomes

The primary outcome was c-AKI, defined by AKI in the presence of postoperative RVF (definition of c-AKI is detailed in Supplemental Table S1). The secondary outcomes were non-congestive AKI, RVF, duration of mechanical ventilation, intensive care unit (ICU) and hospital lengths of stay and in-hospital mortality. AKI was defined by the Acute Kidney Injury Network (AKIN) criteria [26] as >50% relative or >26 μmol/L absolute rise in serum creatinine above preoperative value within 48 h of surgery, or new onset dialysis. Postoperative RVF was defined as new or worsening RVF satisfying published criteria A and/or B [27,28] post separation from CPB and until postoperative day 2 (Supplemental Table S1). Pulmonary artery catheterization is practiced routinely for patients undergoing cardiac surgery at our institution.

- Combined Clinical and Echocardiographic: [28,29]
  - Difficult separation from CPB, characterized by
    1. Concurrent use of ≥1 vasopressor and ≥1 inotrope or ≥1 pulmonary vasodilator (i.e., nitric oxide or epoprostenol); or
    2. More than one CPB weaning attempt; or
    3. Mechanical support device (i.e., RV assist device); and
  - >20% relative reduction in RV fractional area change measured by two-dimensional echocardiography.
- Hemodynamic criteria:
  - CVP >18 mmHg or cardiac index <1.8 L/min/m^2^, in the absence of elevated left atrial and pulmonary capillary wedge pressure >18 mmHg, tamponade, ventricular arrhythmias, or pneumothorax; and
  - RV stroke work index <4 g·min^−1^·m^2^. RVSWI = 0.136 × SVI × (mPAP − RAP); where SVI = stroke volume index = stroke volume/body surface area, mPAP = mean pulmonary artery pressure, and RAP = right atrial pressure.

### 2.3. Cases and Controls

Cases were defined as patients with postoperative RVF, c-AKI or non-congestive AKI. Controls were patients who were free of these complications during the follow up period. Controls were 1:1 matched to the cases based on age and sex. All patients received routine standard of care during the study period.

### 2.4. Study Procedures

All patients were followed prospectively until hospital discharge. Baseline patient characteristics, operative data and postoperative outcomes were recorded by trained research staff. The attending anesthesiologist recorded baseline RV function, and whether difficulty was encountered with CPB separation. Baseline RV function was recorded from the most recent echocardiogram within 90 days of surgery in patients with stable cardiac disease, and during the index surgical admission or intraoperatively prior to CPB in patients who presented acutely.

### 2.5. Biomarkers

Biomarkers were sampled at baseline immediately following anesthesia induction and postoperatively within 1 h of arrival to the ICU. In addition, serum creatinine was measured at least daily during the first 2 postoperative days. Biomarker samples were centrifuged and plasma supernatants stored at −80 °C until analysis. Plasma hs-cTnT (Roche Diagnostics, Indianapolis, IN, USA) and NT-proBNP (Roche Diagnostics) were measured using commercially available US food and Drug Administration-approved electrochemiluminescence immunoassays with a Roche Cobas e411 anaylzer. Manufacturer’s normal reference value for hs-cTnT and NT-proBNP are <13.5 pg/mL (99th percentile) and <300 pg/mL, respectively [23,30]. NGAL levels were measured using a commercially available ELISA kit (Bioporto Diagnostics, Copenhagen, Denmark) and using a mean reference value in healthy volunteers of 35.4 (95% CI, 18.9–46.5) ng/mL [31].

### 2.6. Statistical Analysis

Statistical analyses and graphic representation were performed using SAS 9.4 (SAS Institute, Cary, NC, USA) and Graphpad Prism 6.0 (Graphpad Software, San Diego, CA, USA). Categorical characteristics were compared using *X*^2^ or Fisher’s exact test for categorical variables where appropriate. Continuous variables were compared using Student’s *t* test or Wilcoxon rank sum test depending on normality of distribution. An analysis of variance (ANOVA) was used to compare hospital and ICU stay and ventilation duration between groups. Log transformed NGAL, NT-proBNP, and hs-cTnT were used because their distributions were not normal. A two-way, repeated measure ANOVA was used to compare the effects of time (pre-versus post surgery) and AKI/RVF status for all variables. A Bonferroni correction was used for post hoc pairwise comparison of means. We used conditional logistic regression to assess the association between individual biomarker levels and c-AKI, with and without adjustment for baseline estimated GFR (eGFR), diabetes, and surgery type. These covariates were selected *a priori* based on the strength of their known association with AKI and RVF in cardiac surgical patients [21,32]. As cases were already matched to controls based on age and sex, these covariates were not included in the model. Measure of association was odds ratio (OR) with associated 95% confidence interval (CI). In addition, linear regression was used to assess the association between biomarker levels and continuous outcomes such as length of hospital/ICU stay and duration of mechanical ventilation. The Pearson correlation coefficient was reported as measure of correlation. Area under (AUC) the receiver operator characteristic (ROC) curve was used to assess the ability of individual biomarkers to discriminate between patients who developed each of the outcomes vs. those who did not. Youden’s index was used to identify the optimal ROC cutoff. We also evaluated the incremental value of these biomarkers when added to the clinical model using the method of Delong, Delong, and Clarke-Pearson [33]. *p* < 0.05 was considered statistically significant.

## 3. Results

### 3.1. Patient Characteristics

A total of 350 patients were enrolled in the study, from whom 89 cases were identified and age-sex matched to 89 controls (Supplemental Table S2). Of the cases, 36 (40.5%) developed postoperative RVF, 35 (39.3%) developed non-congestive AKI, and 18 (20.2%) developed c-AKI. Baseline characteristics of c-AKI cases and controls are shown in Table 1. Compared to matched controls, c-AKI patients were more likely to have pre-existing renal insufficiency, diabetes, and to undergo more complex surgery with longer CPB and aortic crossclamp durations. In addition, there was a trend towards higher baseline CVP and lower cardiac index in c-AKI patients. There were no differences in baseline left ventricle ejection fraction (LVEF) between c-AKI and controls.

### 3.2. Biomarker Analysis Pre and Post Cardiac Surgery

**Figure 1:**
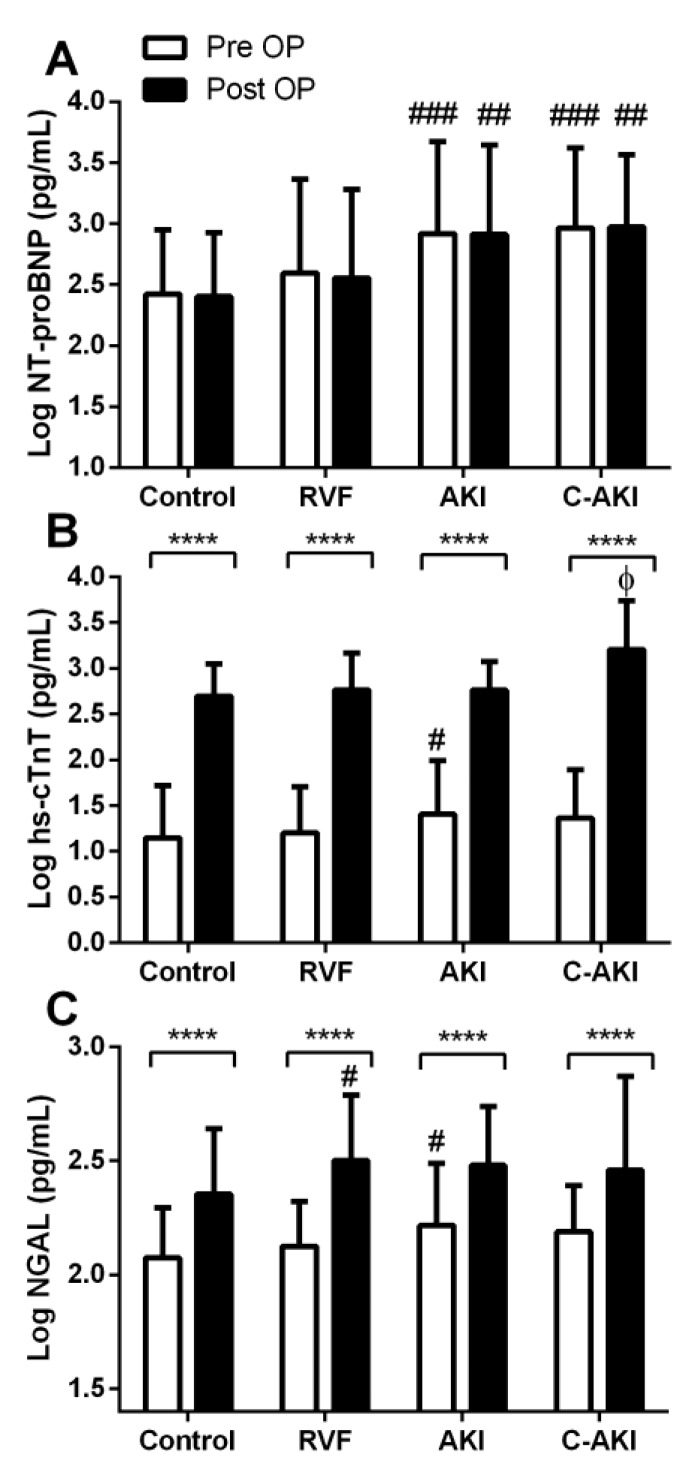
Baseline and postoperative biomarker levels in patients who developed congestive acute kidney injury (c-AKI) vs. controls. **** Within group differences (baseline vs. postoperative) *p* < 0.0001. # Significantly different than control *p* < 0.05, ## *p* < 0.01, ### *p* < 0.001. Φ Significantly different than control, right ventricular failure (RVF), and AKI, *p* < 0.05.

Patients who developed c-AKI had significantly higher baseline NT-proBNP levels compared to controls (Figure 1A). In patients who developed non-congestive AKI, baseline NT-proBNP, NGAL, and hs-cTnT were significantly higher than controls (Figure 1A–C). Following surgery, NT-proBNP remained relatively unchanged while hs-cTnT and NGAL increased 2–2.5 fold in all groups (Figure 1B,C). The magnitude of postoperative increase in hs-cTnT was highest in the c-AKI group. Postoperatively, NGAL increased in those who developed RVF but remained relatively unchanged in the AKI, c-AKI, and control groups.

### 3.3. Hemodynamics Pre and Post Cardiac Surgery

**Figure 2:**
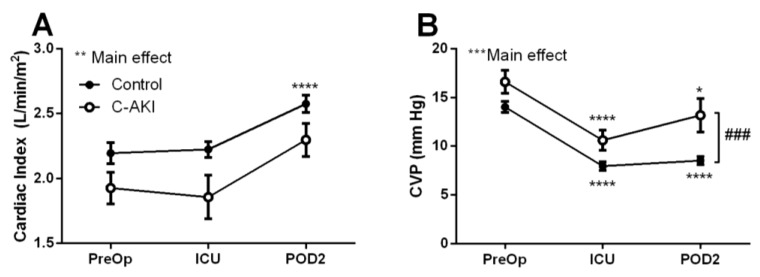
Cardiac index (**A**) and central venous pressure (**B**) in patients who developed congestive acute kidney injury (c-AKI) vs. controls. Hemodynamic variables were assessed preoperatively, upon admission to the intensive care unit and on postoperative day two. * Significantly different than baseline levels, *p* < 0.05; ** *p* < 0.01; *** *p* < 0.001; **** *p* < 0.0001. ### Significant difference between c-AKI and control, *p* < 0.001.

Compared to controls, patients who developed c-AKI had significantly lower cardiac index at all time points (Figure 2A). Conversely, CVP decreased postoperatively from baseline in both c-AKI and control groups but remained higher in c-AKI patients throughout the postoperative period (Figure 2B). This trend was particularly evident on postoperative day 2 where CVP in c-AKI patients remained >1.5 fold higher than in the controls (*p* < 0.001).

In patients with non-congestive AKI, a similar trend was observed where CVP was significantly elevated compared to controls throughout the follow up period (Supplemental Figure S1A). No significant differences in hemodynamic profiles were observed for patients who developed RVF vs. controls (Supplemental Figure S1B,D).

### 3.4. Biomarker and Hemodynamic Correlation

A weak positive relationship was observed between baseline log hs-cTnT and CVP (*r* = 0.21, *p* = 0.006). However, log NT-proBNP and log NGAL levels did not correlate with CVP at baseline. At baseline, cardiac index was inversely correlated with log NT-proBNP (*r* = −0.2, *p* = 0.01) and log hs-cTnT (*r* = −0.19, *p* = 0.01). In addition, there were weak-moderate inverse correlations between baseline eGFR and log hs-cTnT (*r* = −0.27, *p* = 0.0002), log NT-proBNP (*r* = −0.37, *p* < 0.0001), and log NGAL (*r* = −0.28, *p* < 0.0001). Postoperatively, a positive correlation was observed between log NT-proBNP and CVP (*r* = 0.20, *p* = 0.007), but no correlations were found between cardiac index and biomarkers.

### 3.5. Performance of Biomarkers vs. Traditional Parameters for c-AKI Prediction and Detection

#### 3.5.1. Prediction of c-AKI

Higher baseline CVP was associated with c-AKI after multivariable adjustment (adjusted OR 1.20, 95% CI 1.04–1.38 for each 1 mmHg increase in CVP) (Table 2).

**Figure 3:**
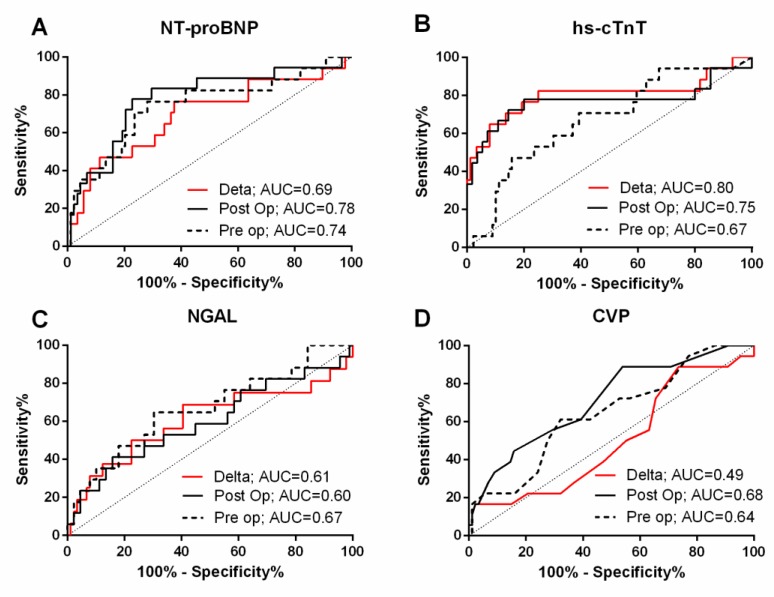
Receiver operating characteristic curves for N-Terminal Pro-B-Type Natriuretic Peptide (NT-proBNP) (**A**), high sensitivity cardiac troponin T (hs-cTnT) (**B**), neutrophil gelatinase-associated lipocalin (NGAL) (**C**), and central venous pressure (**D**). Dotted lines are for baseline assessments, black lines are for postoperative assessments, and red lines are for change in levels (post-preoperative). AUC, area under the curve.

Table 3 and Figure 3 summarize the AUCs for individual biomarkers and traditional parameters for prediction of c-AKI. Of the baseline biomarkers, NT-proBNP had the highest AUC for predicting c-AKI (0.74, 95% CI 0.60–0.89). However, the clinical model alone (based on baseline eGFR, diabetes and surgery type) had an AUC of 0.83 (0.72–0.94), and the addition of NT-proBNP to the clinical model did not significantly improve the AUC (*p* = 0.39, Table 3). The optimal cut-off of baseline NT-proBNP for c-AKI prediction was >476 pg/mL (sensitivity 77%, specificity 72%) (Table 4).

#### 3.5.2. Early Detection of c-AKI

After adjusting for eGFR, diabetes, and type of surgery, postoperative NT-proBNP and hs-cTnT remained robust in detecting c-AKI (Table 2). Of note, postoperative CVP was not associated with c-AKI after risk adjustment.

ROC analysis (Table 3, Figure 3) revealed postoperative NT-proBNP and hs-cTnT as parameters with the highest AUCs for early detection of c-AKI after multivariable adjustment. Specifically, AUC for postoperative NT-proBNP was 0.78 (0.66–0.91) alone (optimal cutoff 599.5 pg/mL, sensitivity 72.2%, specificity 79.5%) and 0.86 (0.76–0.96) after risk adjustment (Table 3). AUC for postoperative hs-cTnT was 0.75 (0.58–0.92) alone (optimal cutoff 1089.0 pg/mL, sensitivity 66.7%, specificity 84.1%) and 0.88 (0.80–0.96) after risk adjustment. Postoperative NGAL alone and CVP alone only yielded moderate AUCs for detecting c-AKI (0.60 for NGAL and 0.68 for CVP).

#### 3.5.3. Perioperative Changes in Biomarker Levels

Change in hs-cTnT levels between baseline and the postoperative period was associated with c-AKI (Table 2 and Table 3). ∆hs-cTnT alone yielded an AUC of 0.8 (0.64–0.96) for c-AKI detection, and was the only biomarker that significantly increased the AUC of the clinical model. Specifically, the AUC was 0.87 (0.76–0.99) when ∆hs-cTnT was combined with baseline creatinine, 0.93 (0.88–0.99) when combined with diabetes status, and 0.94 (0.89–0.99) when combined with the full clinical model (Table 3). The optimal cutoff of ∆hs-cTnT for detecting c-AKI was > 730.9 pg/mL (sensitivity 82%, specificity 75%; Table 4). In contrast, the ability of ∆CVP to detect c-AKI was no better than a coin toss (AUC 0.49, 95% CI 0.34–0.63).

### 3.6. Sample Size Calculation

A post-hoc sample size calculation was performed to validate our findings in light of the size of our cohort. An AUC of 0.70 for biomarker prediction of post cardiac surgery AKI has been deemed clinically meaningful [34]. We conservatively chose a null hypothesis AUC of 0.75. To detect an AUC of 0.93 in our ∆hs-cTnT + diabetes model, our observed 18 c-AKI cases and 89 controls yielded a 86% power using a two-sided *z*-test at an alpha of 0.05.

### 3.7. Secondary Outcomes

Supplemental Table S3 summarizes the AUCs of biomarkers and CVP in predicting non-congestive AKI and postoperative RVF. At baseline, all three biomarkers had moderate accuracy for predicting non-congestive AKI (AUC 0.67 to 0.70). Postoperatively, NT-proBNP detected non-congestive AKI with moderate accuracy (AUC 0.72). ∆Biomarker levels and ∆CVP detected non-congestive AKI poorly (AUC ranging from 0.44–0.57). In contrast, none of the parameters predicted or detected RVF well (AUC 0.48–0.62).

**Figure 4:**
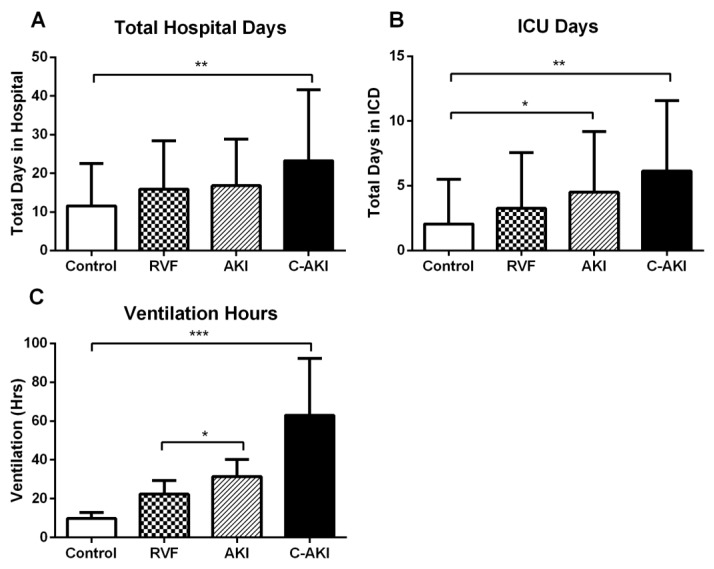
Lengths of stay in hospital (**A**) and ICU (**B**) and duration of mechanical ventilation (**C**) in patients who developed postoperative acute kidney injury (AKI), right ventricular failure (RVF), congestive AKI (c-AKI), and controls. Data expressed mean ± SD. * *p* < 0.05, ** *p* < 0.01, *** *p* < 0.001.

During follow up, 3 (1.7%) patients died (2 c-AKI and 1 control) and 3 patients (1.7%) developed severe AKI requiring renal replacement therapy (2 c-AKI and 1 non-congestive AKI). Compared to controls, patients who developed c-AKI had significantly longer hospitalization, ICU length of stay and duration of mechanical ventilation (Table 1, Figure 4). Non-congestive AKI was associated longer periods of ICU stay and mechanical ventilation compared to controls. In contrast, there were no differences in observed outcomes for patients who developed postoperative RVF alone (Figure 4).

## 4. Discussion

This prospective nested case-control study found cardiac biomarkers hs-cTnT and NT-proBNP to be stronger predictors of c-AKI following cardiac surgery than the renal biomarker NGAL. Four major findings were derived from this study. (1) We identified the novel concept of c-AKI as a cardiorenal syndrome in the context of RVF. C-AKI was associated with prolonged mechanical ventilation and length of stay in hospital and ICU; (2) A single measurement of NT-proBNP or hs-cTnT predicted and detected c-AKI with high accuracy. In contrast, traditional measure of venous congestion such as ∆CVP had an AUC for c-AKI that was analogous to a coin flip. Specifically, baseline and postoperative NT-proBNP yielded AUCs of 0.74 and 0.78, postoperative hs-cTnT yielded an AUC of 0.75, and baseline and postoperative CVP yielded AUCs of 0.64 and 0.68, respectively; (3) ∆hs-cTnT alone yielded an AUC of 0.80 for c-AKI, and the addition of diabetes increased the AUC to 0.93; (4) Neither the cardiorenal biomarkers, clinical variables, nor CVP were able to predict postoperative RVF well.

### 4.1. A New AKI Phenotype

Our study is novel in our definition and characterization of a congestive subtype of AKI. We found that c-AKI was associated with a higher burden of postoperative morbidity and healthcare cost than non-congestive AKI or RVF alone. The etiology of cardiac surgery-associated AKI is multifactorial involving many non-modifiable risk factors such as ischemic-reperfusion injury, inflammation and oxidative stress [14]. Unlike other AKI subtypes, c-AKI is potentially preventable and treatable, although it is often difficult to detect at an early stage. Our findings offer new insights on the role of biomarkers in the prediction and early detection of c-AKI and represent a critical first step towards characterizing postoperative c-AKI. Determining whether a biomarker-guided approach can complement current prediction, prevention and timely management strategies in the perioperative period is an important area for future investigation.

### 4.2. Venous Congestion and CVP

Our study is the first to prospectively characterize postoperative c-AKI with serial cardiac and renal biomarkers. Although several studies have evaluated the utility of similar biomarkers for AKI risk stratification in cardiac surgery cohorts [14,18,21,32,35], none have focused on c-AKI, which is truly a cardiorenal syndrome [15]. AKI in the context of RVF results from a complex series of cardiorenal interactions. This interaction has been believed to be primarily due to renal hypoperfusion [36], but recent evidence suggests venous congestion as a primary contributor [2,15,16,37,38]. Transrenal perfusion pressure is calculated as the mean arterial pressure minus the CVP. Clinically, in patients with acute decompensated heart failure and volume overload, the combination of low systemic pressure with elevated CVP may impair renal perfusion [15,39,40]. In a study of 145 patients admitted with acute decompensated heart failure, and using CVP as a surrogate for systemic venous congestion, Mullens et al. demonstrated that patients with low CVP (<8 mmHg) experienced significantly less decline in renal function compared to those with high CVP (>24 mmHg), independent of their cardiac index [38]. Similarly, we demonstrated that patients who developed postoperative c-AKI had elevated CVP at baseline that persisted into the postoperative period. In further support of a congestive etiology, those who developed c-AKI had LVEFs that were statistically similar to the controls and may benefit from perioperative decongestive strategies. The success of such strategies requires accurate and timely identification of venous congestion, as accumulating evidence suggests a poor correlation between CVP and circulating blood volume and an inability of CVP or ∆CVP to predict fluid responsiveness (ROC AUC value of 0.56) [16,17]. Our study corroborates these findings. Specifically, CVP detected c-AKI poorly (AUC = 0.64 for baseline CVP and AUC = 0.49 for ∆CVP). In contrast, biomarkers of cardiac function and distention (NT-proBNP) and myocardial injury (hs-cTnT) were superior to CVP for predicting and detecting c-AKI.

### 4.3. Congestive AKI and Cardiorenal Biomarkers

NT-proBNP and hs-cTnT are well-established prognosticators in stable and acute decompensated heart failure. NT-proBNP is a prohormone secreted by the atria and ventricles in response to volume and pressure overload. In patients with pulmonary hypertension, NT-proBNP has been shown to increase in proportion to the degree of RV distension and wall stress; whereas hs-cTnT levels increase in proportion to the severity of RV dysfunction [24,25,41]. Several studies have evaluated whether baseline and postoperative BNP could predict cardiac surgery-associated AKI with mixed results (AUC range 0.60 to 0.86) [21,34,35]. To our knowledge, only two studies have evaluated the relationship between hs-cTnT and AKI in the perioperative setting [32,34]. One study reported similar hs-cTnT and NT-proBNP changes in a pediatric cohort to those observed in our adult cohort. In addition, these authors found baseline and postoperative biomarker levels were weakly predictive of AKI (AUC for hs-cTnT: Baseline 0.57, post 0.62; AUC for NT-proBNP: Pre 0.53, post 0.57) [34]. Our study adds to this knowledge by evaluating the same biomarkers to predict RVF, c-AKI, and non-congestive AKI in adult patients. We demonstrate NT-proBNP and hs-cTnT as excellent potential biomarkers for postoperative c-AKI, but poor predictors of isolated RVF. This observation may be explained by two mechanisms. First, potential etiologies for perioperative RVF are diverse, including myocardial ischemia due to poor myocardial preservation [42], graft occlusion, or air emboli [43]. Some of these events are unanticipated complications of CPB and surgery and cannot be predicted using biomarkers or conventional means [44,45]. Second, c-AKI is the end organ manifestation of longer and more severe episodes of perioperative RVF. In our study, there was a positive correlation between hs-cTnT and CPB duration (*r* = 0.44, *p* < 0.0001). Higher postoperative hs-cTnT levels (and thus higher Δhs-cTnT) were likely secondary to complex surgery requiring prolonged CPB with prolonged myocardial ischemia that was possibly compounded by suboptimal myocardial preservation. In addition, prolonged RV ischemia and infarction leading to end organ complications are more likely with complex cardiac procedures. Future studies are needed to fully elucidate mechanisms responsible for hs-cTnT release in c-AKI and congestive states.

### 4.4. Secondary Outcomes

In the cardiac surgical setting, elevated pre and postoperative NT-proBNP and hs-TnT levels have been shown to be associated with prolonged ICU length of stay, mechanical ventilation, and postoperative inotropic support [35,46,47,48,49]. We showed higher preoperative hs-cTnT and postoperative NT-proBNP were associated with increased duration of mechanical ventilation and hospital and ICU stay.

### 4.5. Clinical Implications

Our findings have important implications for the optimization of patients who may be at high-risk for developing postoperative c-AKI. This is especially important, as unlike its non-congestive counterpart, c-AKI, may improve with diuretic, vasodilator therapies, and inotropic support [16,38]. Although both baseline and postoperative NT-proBNP levels were associated with c-AKI, baseline biomarker level may be of greater practical importance as it allows for a greater window of opportunity for preoperative optimization by postponing non-emergent surgery for decongestive therapy. We in addition demonstrated a high AUC of 0.93 (95% CI 0.88–0.99) when combining Δhs-cTnT and diabetes for the early detection of c-AKI in the immediate postoperative period, days before the confirmation of c-AKI by serum creatinine using the traditional AKIN definition. When adjusted for diabetes, each 100 pg/mL increase in Δhs-cTnT is associated with a 23% increased odds of c-AKI (adjusted OR 1.23, 95% 1.10–1.38). In addition, 37% of diabetic patients who had Δhs-cTnT values above the cutoff developed c-AKI, where as none of the non-diabetic patients with Δhs-cTnT values below cutoff developed c-AKI. This simple model helps to efficiently identify a high-risk group that is most likely to benefit from future clinical trials of intensive perioperative monitoring and targeted therapy including fluid restriction, decongestion, and inotropic support. The feasibility of these biomarker-guided trials is enhanced by the availability of NT-proBNP and hs-cTnT as accurate and affordable point of care assays that could be easily and rapidly implemented at the bedside or preoperative assessment clinics [50,51]. Significant progress has been made in the development of these point of care assays, with newer generations demonstrating comparable diagnostic accuracy as high sensitivity core-laboratory assays [50,51].

### 4.6. Study Limitations

This study has several limitations. Firstly, it is single center in nature. However, we recruited a representative sample of patients undergoing all major cardiac surgery, and our sample size was similar to that from other studies in the field. Secondly, c-AKI was a relatively rare event, and the small event rate limited our ability to explore the additive predictive value of biomarkers in more comprehensive clinical models. Thirdly, the number of patients experiencing dialysis or death in our study was low, limiting our ability to evaluate the association of biomarkers with these outcomes.

## 5. Conclusions

Our study findings support hs-cTnT and NT-proBNP as potential biomarkers for prediction of a highly morbid subtype of postoperative AKI that occurs with RVF. Baseline and postoperative NT-proBNP, postoperative hs-cTnT, and ∆hs-cTnT had excellent AUCs for the prediction and early detection of c-AKI. Importantly, the addition of diabetes status to ∆hs-cTnT further increased accuracy for detecting c-AKI. Our findings provide novel insights into cardiorenal physiology in the perioperative setting and may be used to monitor response to goal-directed decongestive therapy in clinical trials to mitigate c-AKI. In addition, the relatively rare incidence of postoperative c-AKI identified in our study highlights the importance of using biomarker models (i.e., ∆hs-cTnT + diabetes) to identify high-risk patients for future interventional studies.

## Figures and Tables

**Table 1:** Characteristics of patients with congestive acute kidney injury (c-AKI) vs. controls.

	Control (*n* = 89)	c-AKI (*n* = 18)	*p*-Value
Baseline Characteristics			
Male	61 (68.5%)	11 (61.1%)	0.54
Age (years)	66.0 (63.8–68.2)	67.1 (61.1–73.2)	0.57
Body Mass Index (kg/m^2^)	28.5 (27.4–29.7)	30.5 (27.3–33.6)	0.35
eGFR (mL/min/1.73 m^2^)	89.8 (83.4–96.1)	75.2 (60.6–89.7)	0.03
Serum Creatinine (µmol/L)	84.3 (79.5–89.1)	100.7 (87.3–114.1)	0.009
Cardiac Index (L/min/m^2^)	2.18 (2.03–2.33)	1.93 (1.66- 2.19)	0.08
Central Venous Pressure (mmHg)	13.9 (12.9–14.9)	16.6 (14.1–19.1)	0.06
Left Ventricle Ejection Fraction (%)	53.1 (51.4–54.9)	49.6 (44.3–54.8)	0.14
Comorbidities			
Hypertension	57 (64%)	12 (67%)	0.83
Diabetes	20 (22%)	12 (67%)	0.0002
COPD	8 (9%)	1 (6%)	1.0
Pre-existing Right Heart Dysfunction	1 (1.1%)	1 (6%)	0.31
Coronary Artery Disease	57 (64%)	11 (61%)	0.81
Medications			
ASA	54 (61%)	11 (62%)	0.97
Beta Blocker	59 (66%)	11 (62%)	0.67
ACE Inhibitor	50 (56%)	8 (44%)	0.36
Lipid Lowering agents	55 (62%)	11 (62%)	0.96
Intraoperative Characteristics			
Surgery Type			0.0006
CABG	43 (48%)	5 (28%)	
Single Valve	33 (37%)	3 (17%)	
Combined CABG/Valve/Other	13 (15%)	10 (56%)	
Cardiopulmonary Bypass Duration (min)	89.1 (82.1–96.0)	141.1 (114.5–167.7)	0.0002
Aortic Cross Clamp Duration (min)	64.6 (58.4–70.8)	102.1 (78.8–125.4)	0.003
Postoperative characteristics			
Length of Hospital Stay (days)	11.8 (9.5–14.1)	23.3 (14.2–32.4)	0.0001
Length of Intensive Care Unit Stay (days)	2.1 (1.3–2.8)	6.1 (3.4–8.8)	<0.0001
Mechanical Ventilation Duration (hours)	9.7 (3.6–15.7)	63.0 (1.23–124.7)	<0.0001

eGFR, estimated glomerular filtration rate; CABG, coronary artery bypass graft; COPD, chronic obstructive pulmonary disease; ASA, acetylsalicylic acid; ACE, angiotensin-converting-enzyme; Data presented are expressed as n (%) or mean (95% confidence interval).

**Table 2:** Association between cardiorenal biomarkers and congestive acute kidney injury.

	Unadjusted		Adjusted ^a^	
	OR (95% CI)	*p* Value	OR (95% CI)	*p* Value
Baseline				
NGAL	1.37 (1.05–1.78)	0.02	1.19 (0.88–1.61)	0.25
NT-proBNP	1.17 (1.06–1.29)	0.001	1.10 (0.97–1.24)	0.12
hs-cTnT	1.06 (0.98–1.15)	0.15	0.99 (0.87–1.11)	0.81
CVP	1.12 (1.01–1.25)	0.03	1.20 (1.04–1.38)	0.01
Postoperative				
NGAL	1.11 (0.93–1.33)	0.25	1.02 (0.83–1.26)	0.86
NT-proBNP	1.21 (1.09–1.34)	0.0005	1.13 (1.00–1.29)	0.05
hs-cTnT	1.26 (1.10–1.45)	0.0009	1.22 (1.05–1.42)	0.008
CVP	1.17 (1.03–1.32)	0.01	1.15 (0.98–1.35)	0.09
Change (Postoperative–Baseline)				
∆NGAL	1.23 (0.96–1.57)	0.09	1.26 (0.93–1.71)	0.13
∆NT-proBNP	0.99 (0.96–1.03)	0.85	0.99 (0.94–1.05)	0.84
∆hs-cTnT	1.15 (1.07–1.24)	0.0002	1.25 (1.09–1.44)	0.001
∆CVP	1.00 (0.91–1.10)	0.94	0.93 (0.83–1.04)	0.20

eGFR, estimated glomerular filtration rate; NGAL, neutrophil gelatinase-associated lipocalin; NT-proBNP, N-Terminal Pro-B-Type Natriuretic Peptide; hs-cTNT, high sensitivity cardiac troponin T; CVP, central venous pressure; OR, odds ratio; CI, confidence interval. ^a^ Biomarker models were adjusted for eGFR, diabetes and type of cardiac surgery. Baseline and postoperative biomarker levels were log transformed. Odds ratios were expressed per 0.1 unit increase in log-transformed baseline and postoperative biomarker levels and per 100 pg/mL increase for ∆biomarker levels.

**Table 3:** Areas under the Receiver-Operating Curve for the prediction of congestive acute kidney injury.

	Unadjusted AUC (95% CI) Biomarker Only	^a^ Adjusted AUC (95%CI) Biomarker + Clinical Model
Clinical Model	-	0.83 (0.72, 0.94)
Baseline		
NGAL	0.67 (0.51–0.82)	0.84 (0.73–0.95)
NT-proBNP	0.74 (0.60–0.89)	0.84 (0.75–0.94)
hs-cTnT	0.67 (0.53–0.81)	0.82 (0.71–0.94)
CVP	0.64 (0.50–0.78)	0.88 (0.80–0.96)
Postoperative		
NGAL	0.60 (0.43–0.77)	0.83 (0.72–0.94)
NT-proBNP	0.78 (0.66–0.91)	0.86 (0.76–0.96)
hs-cTnT	0.75 (0.58–0.92)	0.88 (0.80–0.96)
CVP	0.68 (0.55–0.81)	0.87 (0.80–0.95)
Change (Postoperative-Baseline)		
∆NGAL	0.61 (0.42–0.79)	0.82 (0.72–0.93)
∆NT-proBNP	0.69 (0.54–0.85)	0.83 (0.71–0.94)
∆hs-cTnT	0.80 (0.64–0.96)	* 0.94 (0.89–0.99)
∆CVP	0.49 (0.34–0.63)	0.84 (0.72–0.96)
Combined Model		
∆Hs-cTnT + Preoperative Creatinine	0.87 (0.76–0.99)	-
∆Hs-cTnT + Diabetes	0.93 (0.88–0.99)	-

eGFR, estimated glomerular filtration rate; NGAL, neutrophil gelatinase-associated lipocalin; NT-proBNP, N-Terminal Pro-B-Type Natriuretic Peptide; hs-cTNT, high sensitivity cardiac troponin T; CVP, central venous pressure; AUC, area under the receiver operative characteristic curve; CI, confidence interval. * Significantly improves AUC of the clinical model *p* < 0.05. ^a^ Biomarker models were adjusted for eGFR, type of cardiac surgery and diabetes.

**Table 4:** Cutoff values for predicting congestive acute kidney injury.

	Cutoff	Sensitivity	Specificity
Baseline			
NGAL (pg/mL)	140.2	64.7	69.7
NT-proBNP (pg/mL)	476.0	76.5	71.9
hs-cTnT (pg/mL)	25.0	47.1	84.3
CVP	15.5	61.1	67.8
Postoperative			
NGAL (pg/mL)	440.9	41.2	84.3
NT-proBNP (pg/mL)	599.5	72.2	79.5
hs-cTnT (pg/mL)	1089.0	66.7	84.1
CVP	10.5	44.4	84.3
Change (Postoperative-Baseline)			
∆NGAL (pg/mL)	181.7	50.0	77.5
∆NT-proBNP (pg/mL)	−38.8	76.5	62.5
∆hs-cTnT (pg/mL)	730.9	82.4	75.0
∆CVP	−9.5	88.9	26.4

NGAL, neutrophil gelatinase-associated lipocalin; NT-proBNP, N-Terminal Pro-B-Type Natriuretic Peptide; hs-cTNT, high sensitivity cardiac troponin T; CVP, central venous pressure.

## Supplementary Materials

The following are available online at http://www.mdpi.com/2077-0383/7/12/540/s1.
