# Supplementary material for: Utility of Novel Cardiorenal Biomarkers in the Prediction and Early Detection of Congestive Kidney Injury Following Cardiac Surgery
Source: J Clin Med. 2018 Dec 12;7(12):540. doi: 10.3390/jcm7120540 (PMC6306702; doi:10.3390/jcm7120540)
Supplement: Supplementary file 1 [file jcm-07-00540-s001.pdf]

Supplemental Material

Supplemental Table 1. Diagnostic criteria for primary and secondary outcomes

Non-Congestive AKI	Right Ventricular Failure[1,2]	Congestive AKI
(AKIN) criteria:<br>1) >50% relative or >26 μmol/L absolute rise in serum creatinine above preoperative value within 48 hours of surgery<br><br>OR<br>2) New onset dialysis within 48 hours of surgery	***A. Combined Clinical and Echocardiographic:***<br>i. Difficult separation from CPB, characterized by<br>1. Concurrent use of ≥1 vasopressor and ≥1 inotrope or ≥1 pulmonary vasodilator (i.e. nitric oxide or epoprostenol); or<br>2. More than one CPB weaning attempt; or<br>3. Mechanical support device (i.e., RV assist device); and<br>ii. >20% relative reduction in RV fractional area change measured by two-dimensional echocardiography.<br><br>AND/OR<br>***B. Hemodynamic criteria:***<br>i. CVP >18 mmHg or cardiac index < 1.8 L/min/m^2, in the absence of elevated left atrial and pulmonary capillary wedge pressure >18 mmHg, tamponade, ventricular arrhythmias or pneumothorax; and<br>ii. RV stroke work index <4 g.min^{-1}.m^2. RVSWI = 0.136 x SVI x (mPAP – RAP).	Meets criteria for non-Congestive AKI and RVF

AKI, acute kidney injury; RVF, right ventricular failure; CVP, central venous pressure; RVSWI, right ventricle stroke work index; SVI, Stroke Volume Index (stroke volume/body surface area); mPAP, mean pulmonary artery pressure; RAP, right atrial pressure

Supplemental Table 2. Characteristics of cases vs. controls.

	Control (n=89)	C-AKI (n=18)	AKI (n=35)	RHF (n=36)	P-Vale
Baseline Characteristics					
Male	61 (68.5%)	11 (61.1%)	25 (71.1%)	25 (69.4%)	0.89
Age (years)	66.0 (63.8-68.2)	67.1 (61.1-73.2)	68.5 (65.2—71.7)	67.4 (63.9-70.7)	0.69
Body Mass Index (kg/m^2)	28.5 (27.4-29.7)	30.5 (27.3-33.6)	33.0 (26.9-39.1)	29.6 (27.8- 31.5)	0.14
eGFR (mL/min/1.73m^2)	89.8 (83.4-96.1)	75.2 (60.6-89.7)	82.4 (71.4-93.4)	89.9 (76.0-103.7)	0.29
Serum Creatinine (μmol/L)	84.3 (79.5-89.1)	100.7 (87.3-114.1)	102.5 (86.8-118.3)	90.1 (80.0-100.2)	0.02
Cardiac Index (L/min/m^2)	2.18 (2.03-2.33)	1.93 (1.66- 2.19)	2.11 (1.93-2.28)	2.14 (1.92-2.36)	0.52
Central Venous Pressure (mmHg)	13.9 (12.9-14.9)	16.6 (14.1-19.1)	15.8 (14.1-17.5)	15.3 (14.0-16.6)	0.06
Left Ventricle Ejection Fraction (%)	53.1 (51.4-54.9)	49.6 (44.3-54.8)	50.4 (46.3-54.5)	51.4 (47.8-55.1)	0.37
Comorbidities					
Hypertension	57 (64%)	12 (67%)	27 (71%)	26 (72%)	0.55
Diabetes	20 (22%)	12 (67%)	18 (51%)	17 (47%)	0.0002
COPD	8 (9%)	1 (6%)	5 (14%)	6 (17%)	0.49
Pre-existing Right Heart Dysfunction	1 (1.1%)	1 (6%)	0 (0%)	1 (2.8%)	0.45
Coronary Artery Disease	57 (64%)	11 (61%)	22 (62%)	23 (64%)	0.99
Medications					
ASA	54 (61%)	11 (62%)	23 (66%)	26 (72%)	0.66
Beta Blocker	59 (66%)	11 (62%)	29 (83%)	25 (69%)	0.26

ACE Inhibitor	50 (56%)	8 (44%)	24 (69%)	19 (53%)	0.34
Lipid Lowering agents	55 (62%)	11 (62%)	26 (74%)	27 (75%)	0.36
Intraoperative Characteristics					
Surgery Type					
CABG	43 (48%)	5 (28%)	12 (34%)	11 (31%)	0.005
Single Valve	33 (37%)	3 (17%)	10 (29%)	11 (31%)	
Combined CABG/Valve/Other	13 (15%)	10 (56%)	13 (37%)	14 (39%)	
Cardiopulmonary Bypass Duration (min)	89.1 (82.1-96.0)	141.1 (114.5-167.7)	116.8 (99.7-133.9)	114.4 (100.5-128.3)	<0.0001
Aortic Cross Clamp Duration (min)	64.6 (58.4-70.8)	102.1 (78.8-125.4)	82.1 (69.2-95.0)	87.4 (74.3-100.4)	<0.0001
Postoperative characteristics					
Length of Hospital Stay (days)	11.8 (9.5-14.1)	23.3 (14.2-32.4)	17.4 (13.4-21.4)	16.1 (12.0-20.3)	0.002
Length of Intensive Care Unit Stay (days)	2.1 (1.3-2.8)	6.1 (3.4- 8.8)	4.7 (3.1-6.2)	3.3 (1.9-4.7)	0.0002
Mechanical Ventilation Duration (hours)	9.7 (3.6-15.7)	63.0 (1.23-124.7)	32.9 (15.0-50.9)	22.8 (8.7-36.8)	0.001

eGFR, estimated glomerular filtration rate; CABG, coronary artery bypass graft; COPD, chronic obstructive pulmonary disease; ASA, Acetysalicylic acid (Aspirin); ACE, angiotensin-converting-enzyme.

Supplemental Table 3 ROC analysis: Area under the Receiver-Operating Characteristic curve values for the prediction of non-congestive acute kidney injury and right ventricular failure.

	**Non-Congestive AKI AUC (95% CI)**	**Right Ventricular Failure AUC (95% CI)**
**Baseline**		
NGAL	0.67 (0.56-0.78)	0.55 (0.43-0.66)
NT-proBNP	0.70 (0.58-0.82)	0.57 (0.45-0.69)
hs-cTnT	0.68 (0.56-0.79)	0.54 (0.43-0.65)
CVP	0.60 (0.48-0.71)	0.60 (0.49-0.70)
**Postoperative**		
NGAL	0.63 (0.53-0.74)	0.63 (0.52-0.74)
NT-proBNP	0.72 (0.60-0.84)	0.60 (0.44-0.69)
hs-cTnT	0.56 (0.45-0.67)	0.53 (0.41-0.64)
CVP	0.60 (0.49-0.71)	0.48 (0.37-0.59)
**Change (Postoperative - Baseline)**		
ΔNGAL	0.53 (0.41-0.66)	0.62 (0.51-0.74)
ΔNT-proBNP	0.44 (0.30-0.58)	0.53 (0.41-0.65)
Δhs-cTnT	0.57 (0.45-0.68)	0.52 (0.40-0.64)
ΔCVP	0.54 (0.42-0.65)	0.58 (0.48-0.68)

CVP, central venous pressure; neutrophil gelatinase-associated lipocalcin, NGAL; N-Terminal Pro-B-Type Natriuretic Peptide, NT-proBNP; hs-cTNT, high sensitivity cardiac troponin T

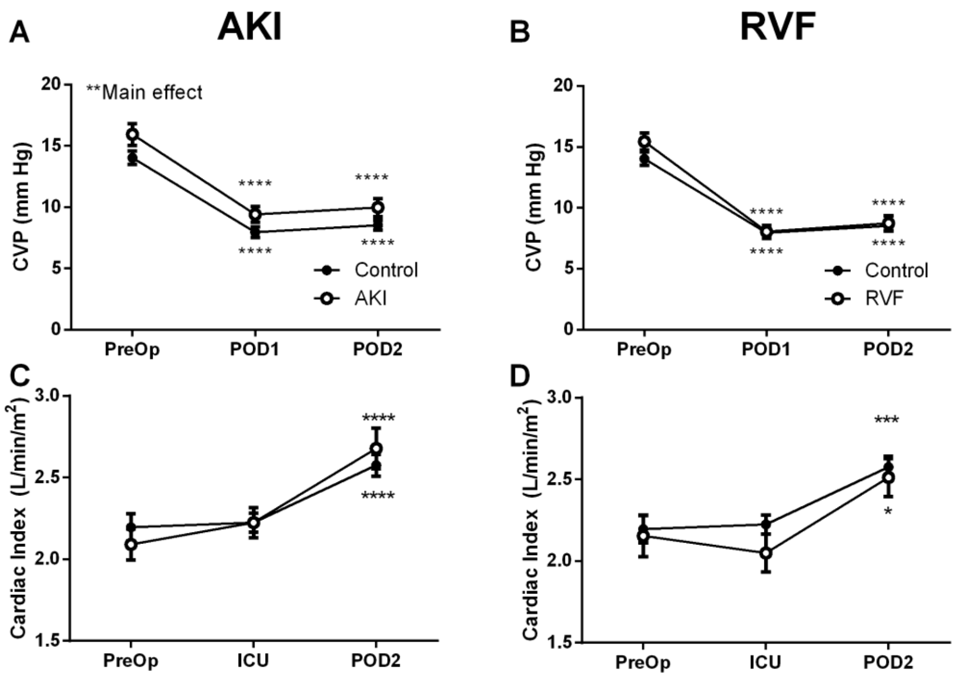

Supplemental Figure 1. Central venous pressure (A,B), and cardiac index (C/D), in patients who developed non-congestive acute kidney injury (AKI) or right ventricular failure (RVF) vs. controls. Hemodynamic variables were assessed preoperatively, upon admission to ICU and on postoperative day two. * Significantly different than preoperative levels, P<0.05; **P<0.01; ***P<0.001; **** P<0.0001.

References

1. Denault AY, Bussières JS, Arellano R, et al. A multicentre randomized-controlled trial of inhaled milrinone in high-risk cardiac surgical patients. *Can J Anaesth*. 2016;63(10):1140-1153. doi:10.1007/s12630-016-0709-8

2. Rudski LG, Lai WW, Afilalo J, et al. Guidelines for the echocardiographic assessment of the right heart in adults: a report from the American Society of Echocardiography endorsed by the European Association of Echocardiography, a registered branch of the European Society of Cardiology, and the Canadian Society of Echocardiography. *J Am Soc Echocardiogr*. 2010;23(7):685-713; quiz 786-788. doi:10.1016/j.echo.2010.05.010
